# AAA237, an SKP2 inhibitor, suppresses glioblastoma by inducing BNIP3-dependent autophagy through the mTOR pathway

**DOI:** 10.1186/s12935-023-03191-3

**Published:** 2024-02-10

**Authors:** Yizhi Zhang, Wan Li, Yihui Yang, Sen Zhang, Hong Yang, Yue Hao, Xu Fang, Guanhua Du, Jianyou Shi, Lianqiu Wu, Jinhua Wang

**Affiliations:** 1 The State Key Laboratory of Bioactive Substance and Function of Natural Medicines, Beijing, 100050 China; 2 Key Laboratory of Drug Target Research and Drug Screen, Institute of Materia Medica, Chinese Academy of Medical Science and Peking Union Medical College, Beijing, 100050 China; 3 Department of Pharmacy, Personalized Drug Therapy Key Laboratory of Sichuan Province, Sichuan Academy of Medical Sciences & Sichuan Provincial People’s Hospital, School of Medicine, University of Electronic Science and Technology of China, Chengdu, 610072 Sichuan China; 4 Department of Pharmacology, Institute of Materia Medica, Chinese Academy of Medical Science and Peking Union Medical College, Beijing, 100050 China

**Keywords:** Glioblastoma, AAA237, BNIP3, Autophagosome–lysosome fusion

## Abstract

**Background:**

Glioblastoma (GBM) is the most common brain tumor with the worst prognosis. Temozolomide is the only first-line drug for GBM. Unfortunately, the resistance issue is a classic problem. Therefore, it is essential to develop new drugs to treat GBM. As an oncogene, Skp2 is involved in the pathogenesis of various cancers including GBM. In this study, we investigated the anticancer effect of AAA237 on human glioblastoma cells and its underlying mechanism.

**Methods:**

CCK-8 assay was conducted to evaluate IC_50_ values of AAA237 at 48, and 72 h, respectively. The Cellular Thermal Shift Assay (CETSA) was employed to ascertain the status of Skp2 as an intrinsic target of AAA237 inside the cellular milieu. The EdU-DNA synthesis test, Soft-Agar assay and Matrigel assay were performed to check the suppressive effects of AAA237 on cell growth. To identify the migration and invasion ability of GBM cells, transwell assay was conducted. RT-qPCR and Western Blot were employed to verify the level of BNIP3. The mRFP-GFP-LC3 indicator system was utilized to assess alterations in autophagy flux and investigate the impact of AAA237 on the dynamic fusion process between autophagosomes and lysosomes. To investigate the effect of compound AAA237 on tumor growth in vivo, LN229 cells were injected into the brains of mice in an orthotopic model.

**Results:**

AAA237 could inhibit the growth of GBM cells in vitro. AAA237 could bind to Skp2 and inhibit Skp2 expression and the degradation of p21 and p27. In a dose-dependent manner, AAA237 demonstrated the ability to inhibit colony formation, migration, and invasion of GBM cells. AAA237 treatment could upregulate BNIP3 as the hub gene and therefore induce BNIP3-dependent autophagy through the mTOR pathway whereas 3-MA can somewhat reverse this process. In vivo*,* the administration of AAA237 effectively suppressed the development of glioma tumors with no side effects.

**Conclusion:**

Compound AAA237, a novel Skp2 inhibitor, inhibited colony formation, migration and invasion of GBM cells in a dose-dependent manner and time-dependent manner through upregulating BNIP3 as the hub gene and induced BNIP3-dependent autophagy through the mTOR pathway therefore it might be a viable therapeutic drug for the management of GBM.

**Graphical Abstract:**

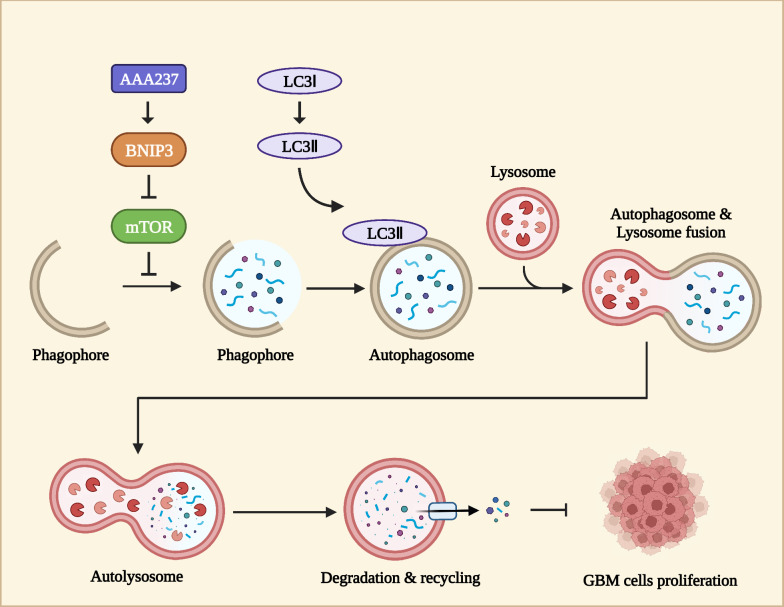

**Supplementary Information:**

The online version contains supplementary material available at 10.1186/s12935-023-03191-3.

## Introduction

Due to its aggressive and invasive nature, Glioblastoma continues to be the prevailing primary malignant neoplasm affecting the adult brain [1]. The treatment approach for glioblastoma includes surgical intervention as well as adjuvant chemotherapy and radiation. It is well recognized that surgery has paramount significance in determining the prognosis of individuals afflicted with this condition [2, 3]. No pharmacological intervention has been shown to change the course of the disease, except for the prolonged progression-free survival offered by the vascular endothelial growth factor antibody, bevacizumab, but not overall survival [4]. Since the registration trial of temozolomide, there has been no substantial improvement in chemotherapy for patients with newly diagnosed glioblastoma [5]. Studies showed that adding temozolomide chemotherapy to standard radiotherapy (60 Gy for 6 weeks) improved survival in patients aged 70 years or younger [6]. However, due to the heterogeneity of the tumor microenvironment, infiltration of glioma stem cells, and low immunogenicity, GBM is characterized by a tendency to develop resistance to radiotherapy, recurrence and a low immune response [7]. Although therapeutic advances have reached increasing improvements in shorter-term survival rates, GBM patients have remained a poor prognosis. Therefore, more effective medicines to enhance the prognosis of GBM patients are urgently needed.

Autophagy is a cellular mechanism in which a cell selectively sequesters its cytoplasmic proteins or organelles, enclosing them within vesicles that subsequently merge with lysosomes. This fusion results in the formation of autophagic lysosomes, which break down the encapsulated contents. Through this process, the cell effectively meets its metabolic requirements and facilitates the regeneration of certain organelles [7, 8]. Aberrant autophagy has been implicated in various diseases including glioblastoma [9]. At the benign stage, autophagy has been shown to perform a tumor-suppressive role, while faulty autophagy has been linked to DNA damage and cancer [10, 11]. Autophagy performs multiple functions in tumor formation and progression. The process of autophagy serves as a mechanism for suppressing tumor formation in the development of cancer by maintaining the stability of cellular and genomic conditions [12]. However, to deal with various biological stresses, tumor cells’ cytoprotective autophagy increases tumor progression [13]. Recently, targeting autophagy as a potential therapy for GBM has been proposed. Inhibition of protective autophagy can make GBM cells more sensitive to chemotherapeutic or radiotherapeutic agents, but excessive autophagic activation in GBM cells can also induce autophagic cell death [14]. Diverse autophagy modulators, such as CQ, HCQ, and Lys05, have been evaluated for their anti-GBM efficacy [15].

Several approved, experimental pharmaceuticals and natural compounds were reported to induce autophagy in various types of cancer [16–18]. Metformin was associated with a 30% reduction in cancer occurrence, according to retrospective data analyses from patients with type 2 diabetes (T2D) [19, 20]. Metformin can enhance TRAIL-induced cell death in TRAIL-resistant lung cancer cells by activating the autophagy flux, as demonstrated by a dose-dependent accumulation of LC3-II and a decrease in the p62 protein levels [16]. Quercetin (3,5,7,3′,4′-Pentahydroxyflavone), among many other naturally occurring substances, has been recognized as an autophagy inducer [21–23]. A preliminary investigation shown that the administration of quercetin resulted in the stimulation of acidic vesicular organelles and autophagic vacuoles, leading to an increase in the ratio of LC3-II/LC3-I. Additionally, quercetin facilitated the recruitment of LC3-II to the autophagosomes, therefore initiating autophagy in cells affected by gastric cancer [24].

In this study, the effect of AAA237 on human glioblastoma cells and its underlying mechanism were investigated. AAA237 dose-dependently inhibited the proliferation of human glioblastoma cells U251 and LN229 with IC_50_ values of 0.485 and 0.407 μM at 48 h, respectively. AAA237 could significantly inhibit the process of growth, migration, invasion, and colony formation in glioblastoma multiforme cells. In addition, AAA237 can upregulate the level of hub gene, BNIP3, proved to downregulate the mTOR pathway, thereby activating autophagy. Meanwhile, analysis of differential expression genes (DEGs) enrichment and pathway enrichment revealed that AAA237 would exert anti-glioblastoma effects by regulating the mTOR pathway. Furthermore, AAA237 could enhance the dynamic fusion process between autophagosomes and lysosomes. In vivo, AAA237 significantly inhibited the tumorigenicity in the LN229 orthotopic model with no significant adverse effects on the organism. All the above results suggest that AAA237 might be a promising drug in the remedy of glioblastoma.

## Materials and methods

### Cell proliferation assay

Cells were seeded in the 96-well plate at a density of 4 × 10^3^ cells/well and cultured at 37 °C with 0, 0.03, 0.1, 0.3, 1, 3, 10, 30, 100 μM AAA237 for 48 h and 72 h. After treatment, 10 μL of CCK-8 reagent was added into each well and incubated for 1 h. Then A450 was measured.

### Cellular thermal shift assay (CETSA)

The supernatants of U251 and LN229 cell lysates were divided into two equal portions, one of which was used as the control group (DMSO), and the other was used as the drug incubation group and incubated at room temperature for 2 h. Then the two supernatants were divided into six equal portions, and were heated for 10 min at 45, 50, 55, 60, 65, and 70 °C, respectively, and cooled, and the cooled supernatants were added with loading buffer and were heated at 70 °C for 10 min, and then the SKP2 expression was detected by Western blot at the end of the process.

### EdU-DNA synthesis assay

U251 and LN229 cells were inoculated in 96-well plates at a density of 4000 per well, and after incubation until the second day, the experimental group incubated the cells with AAA237 at concentrations of 0.3, 1, and 3 μM for 48 h and 72 h, respectively, and the control group was incubated with DMSO. The cells were then stained with EdU, Apollo 567, Hoechst 33342 and finally photographed with a fluorescence microscope (Nikon Eclipse Ti-U) following the steps in the kit.

### Colony formation assay and 3D matrigel culture

About 3000 U251 and LN229 cells were put into six-well plate. After continuously being treated with AAA237 at 0, 0.3, 1, 3 μM for 2 weeks. After being washed, the clones were photographed. To do the 3D-matrigel experiment, the lower layer of gel was laid down first, and after waiting for about 20 min for the lower layer to solidify, the upper layer containing about 3000 U251 and LN229 cells was added. After 3–4 weeks of incubation, the formation of cell spheres can be observed under the microscope.

### Transwell assay

To determine the ability of the cells to migrate and invade after the administration of AAA237, 24-well plates with transwell chambers with an 8 μm pore size (Corning Costar, USA) were used. 400 μL of the suspension containing 2 × 10^5^ cells/mL cells was added to the upper chamber, and the plates were incubated for 3–4 h (the invasion required pre-spreading of Matrigel Matrix with pre-cooled serum-free and culture medium in a 1:7 ratio in the upper chamber). Once the cells were attached to the bottom, the culture medium was gently aspirated off of the upper chamber, and 200–300 μL of serum-free culture was added. 600 μL of 1640 culture medium containing 10–20% FBS was added to the lower chamber, and the plates were incubated for 19 (migration) or 24 h (invasion). After the incubation, the cells that successfully traversed the polycarbonate membrane were subjected to fixation using a 4% paraformaldehyde solution (G1101, Servicebio, China) for a duration of 15 min. Subsequently, these fixed cells were stained with a 1% crystal violet solution for a period of 30 min. The cells were visualized using a microscope manufactured by Nikon, a company based in Tokyo, Japan. Five fields per chamber were assessed for the numbers of migrated/invaded cells.

### Western blot

Supernatants from the cell lysates were obtained by RIPA after being centrifuged. The blots were subsequently subjected to blocking in a 5% fat-free milk solution for a duration of 2 h at room temperature, followed by gentle agitation overnight at 4 °C with the primary antibodies. The principal antibodies used in this study were Actin (Proteintech, Rosemont, USA), SKP2, P27, P21, BNIP3, p-mTOR, mTOR, P62, Beclin1, ATG5, and LC3II (Cell Signalling Technology, Danvers, USA) (1000x dilution) (the detailed information of antibodies was shown in Additional file 1: Table S1). The membranes were cut horizontally. After being washed, the blots underwent incubation with the appropriate HRP-linked secondary antibody (Cell Signaling, Danvers, USA). In this research, the P62 protein was stripped and re-probed after being probed of Beclin1, the P27 protein was stripped and re-probed after being probed of P21, the mTOR protein was stripped and re-probed after being probed of p-mTOR.

### RNA sequencing

Next-generation sequencing was performed by Expandbiotech Corporation (Beijing, China) on cells lysed in TRIzol. Using the DESeq2 R package (1.20.0), differential expression analysis was conducted.

### RNA extraction and RT-qPCR

TRIzol Reagent was used to get total RNA, and a PrimeScript RT Reagent Kit was used to make cDNA. Real-time RT-PCR was used with the SYBR Premix Ex TaqIIKit to find the expression of differently expressed genes (the detailed information of sequence of primers was shown in Additional file 1: Table S2).

### RNA interference

Based on the BNIP3 gene sequence and the principles of small molecule interfering RNA (siRNA) design, we designed and synthesized an siRNA targeting the human BNIP3 gene, 5ʹ-AAGGAACACGAGCGUCAUGAAdTdT-3ʹ. Logarithmic growth phase cells were selected and transfected with liposomal lipofectamin 3000-mediated interfering chain BNIP3 siRNA for U251 and LN229, respectively. After 48 h of transfection, mTOR protein level was measured by Western Blot.

### Autophagic flux analysis

As per the guidelines provided by the manufacturer, the cells were subjected to infection using a tandem mRFP-GFP-LC3 adenovirus. Briefly, 5×10^4^ U251 and LN229 cells were planted on a Glass Bottom Cell Culture Dish (801002, NEST, China) and infected for 24 h with tandem mRFP-GFP-LC3 adenovirus (1×10^8^ TU/mL) at MOI = 1. Laser confocal microscopy was used to examine cells after they had been infected. Autophagosomes are shown by yellow puncta, whereas autolysosomes are represented by red puncta.

### Transmission electron microscopy

Cells were fixed for 30 min at room temperature using an electron microscope fixative (G1102, Servicebio, China) and then postfixed for 2 h with 1% OsO4. After being dehydrated in gradient ethanol, the samples were infiltrated and embedded in epoxy resin (ZXBR, Spon 812). A transmission electron microscope (HITACHI HT7800; Tokyo, Japan) was used to photograph the ultrastructure of U251 and LN229 cells.

### Xenograft GBM models In vivo

#### Animals

The female BALB/c-nude mice (17–19 g) were purchased from Beijing Vital River Laboratory (Beijing, China). Prior approval for these experiments was obtained from the ethics committee for laboratory animal care and the Institute of Materia Medica, Chinese Academy of Medical Science and Peking Union Medical College, located in Beijing, China.

#### LN229 glioma orthotopic model

Mice were put into sleep by injecting them with 0.2 mL of 0.6% sodium pentobarbital. A hole was made into the head 3 mm to the right and 0.5 mm in front of the bregma. The LN229 cells were taken out and put back into phosphate-buffered saline (PBS) until there were 5 × 10^6^ cells/mL. The needle was inserted into the hole until it reached a depth of 3.3 mm, corresponding to the location of the right striatum. Cells was injected into the designated region at a controlled rate of 1 μL per minute, followed by the gradual withdrawal of the needle after a duration of 5 min. Then, bone wax was used to cover the pinhole.

### Statistical analysis

The results are reported in the form of mean ± standard deviation (SD). Statistical significance between two groups or more than two groups was performed by Unpaired student’s t-test or one-way-ANOVA using GraphPad Prism 8.0 (GraphPad Software Inc., San Diego, CA, USA), and *P* < 0.05 was considered significant.

## Results

### AAA237 reduced viability and inhibited the proliferation of GBM cells in a dose- and time-dependent manner

**Fig. 1:**
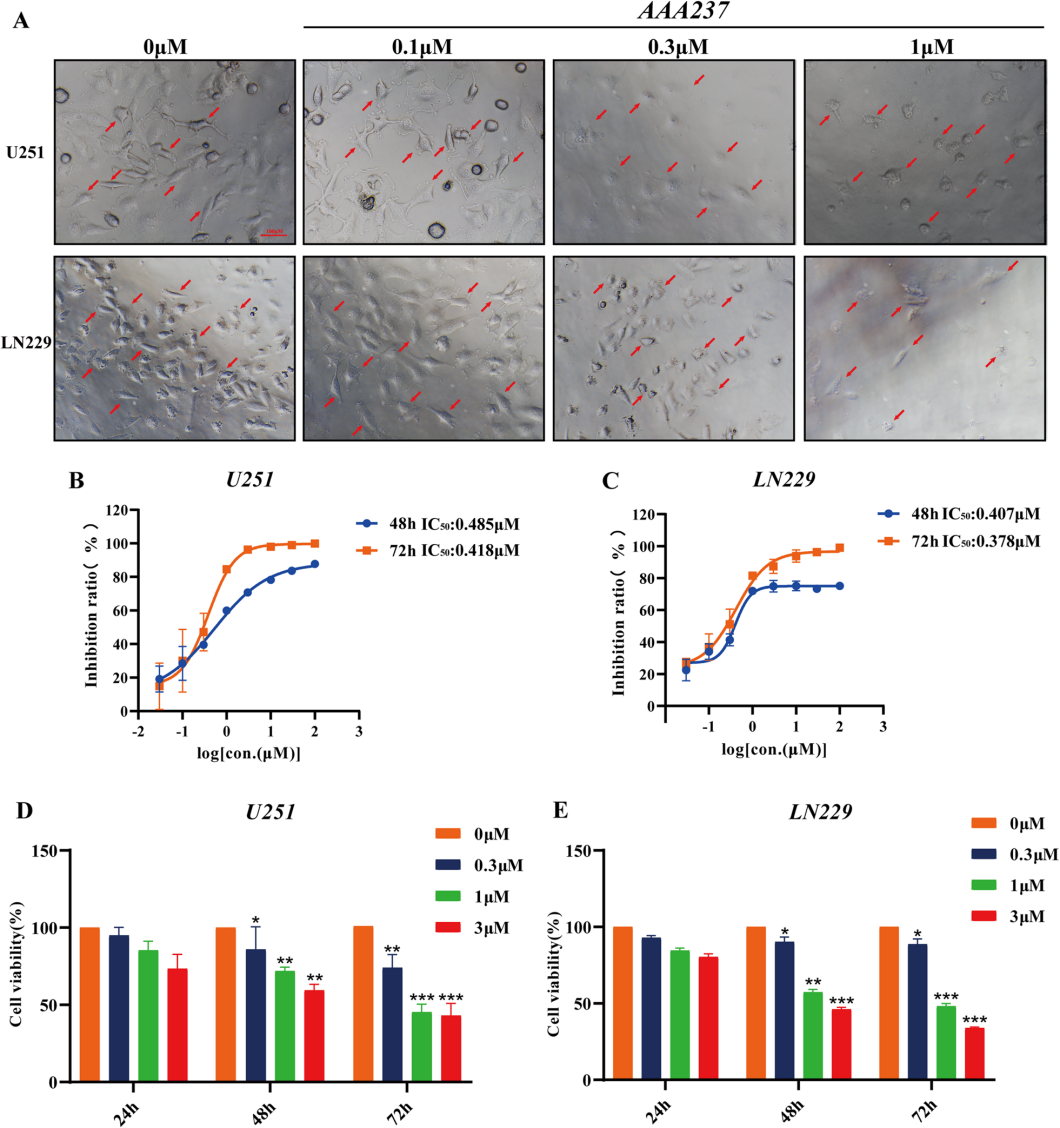
AAA237 suppressed viability and inhibited the proliferation of GBM cells in a dose- and time-dependent manner. **A** After incubation with different concentrations (0, 0.1, 1 and 3 μM) of AAA237 for 48 h, the changes in cell morphology were imaged. Scale bar = 100 μm. IC50 of AAA237 on U251 (**B**) and LN229 cells (**C**) at 48 and 72 h. CCK8 assay shows that AAA237 inhibits proliferation of U251 (**D**) and LN229 (**E**) cells

The changes in cell morphology were imaged to determine the effect of AAA237 on the viability and proliferation of GBM cells. At the 48-h time point, the administration of AAA237 resulted in a dose-dependent alteration in the morphology of U251 and LN229 cells (Fig. 1A). The IC_50_ values for AAA237 on U251 cells at 48 h and 72 h were 0.485 μM, and 0.418 μM; and for LN229 cells, 0.407 μM, and 0.378 μM at 48, and 72 h (Fig. 1B, C) respectively. In order to get a deeper understanding of the effects of AAA237 on the survival and growth of glioblastoma multiforme cells, a CCK-8 experiment was carried out. The findings of the study revealed that the compound AAA237 had a suppressive impact on the growth of U251 and LN229 cells, with the degree of inhibition being dependent on both the dosage and duration of treatment (Fig. 1D, E). Altogether, the results demonstrated that AAA237 could inhibit the growth of GBM cells in vitro.

### AAA237 bound to Skp2 and inhibited Skp2 substrate degradation

Skp2, a well-studied Skp2-SCF E3 ligase complex member, can attach K48-linked and K63-linked ubiquitin chains to a wide variety of substrates [25]. In several types of human cancer, high levels of Skp2 expression are linked to poor prognosis and unfavorable therapy outcomes [26]. AAA237 has apparent interaction with Skp2 protein. The Cellular Thermal Shift Assay (CETSA) assay, which utilizes the generation of a melting curve for a specific protein in cell lysates, was employed to ascertain the status of Skp2 as an intrinsic target of AAA237 inside the cellular milieu. The melting temperature (Tm) of a protein is significantly altered when a compound like AAA237 binds to it.

**Fig. 2:**
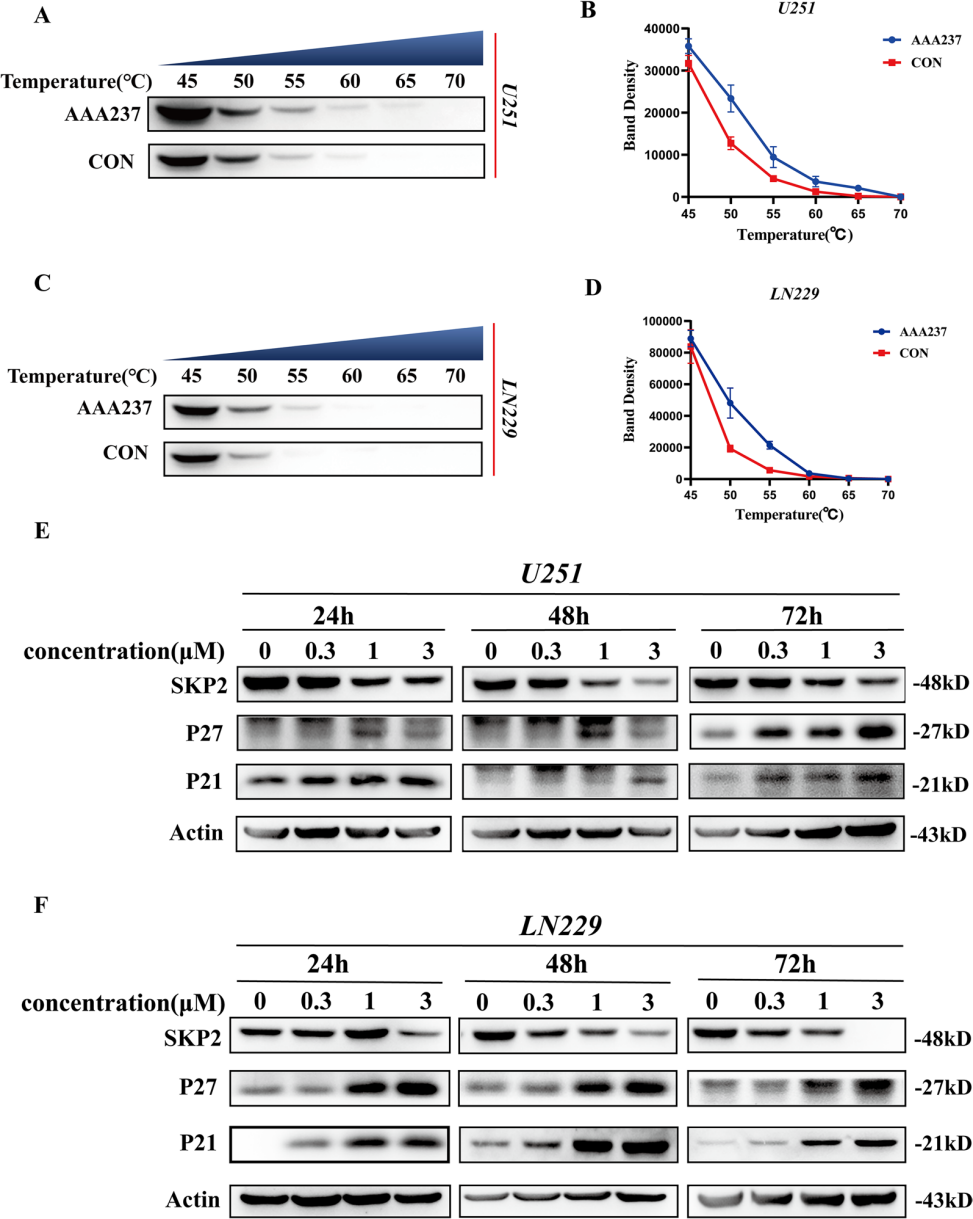
The binding of AAA237 to Skp2 inhibited Skp2 substrate degradation. Cellular thermal shift assay of Skp2 with AAA237 in U251 (**A**, **B**) and LN229 (**C**, **D**). Western blot analysis for protein levels of Skp2, p21Cip1 and p27Kip1 in U251 (**E**) and LN229 (**F**) cells after treatment of AAA237

The experimental results demonstrated that AAA237 exhibited a notable enhancement in the thermal stability of Skp2 compared to the control group in U251 and LN229 cell lines, indicating that it binds directly to intracellular Skp2 (Fig. 2A–D). Subsequently, an examination was conducted to determine the possible impact of AAA237 on the protein expression of Skp2. The results shown that AAA237 exhibited a time-dependent inhibition of Skp2 protein expression in U251 and LN229, and the experimental intervention of AAA237 resulted in an upregulation of the expression levels of p21 and p27 in U251 and LN229 cell lines (Fig. 2E, F). These two proteins, p21 and p27, are well recognized as prominent substrates of Skp2.

Collectively, based on these findings, the binding of AAA237 to Skp2 would inhibit Skp2 expression and the degradation of p21 and p27.

### AAA237 inhibited colony formation, migration and invasion of GBM cells in a dose-dependent manner

**Fig. 3:**
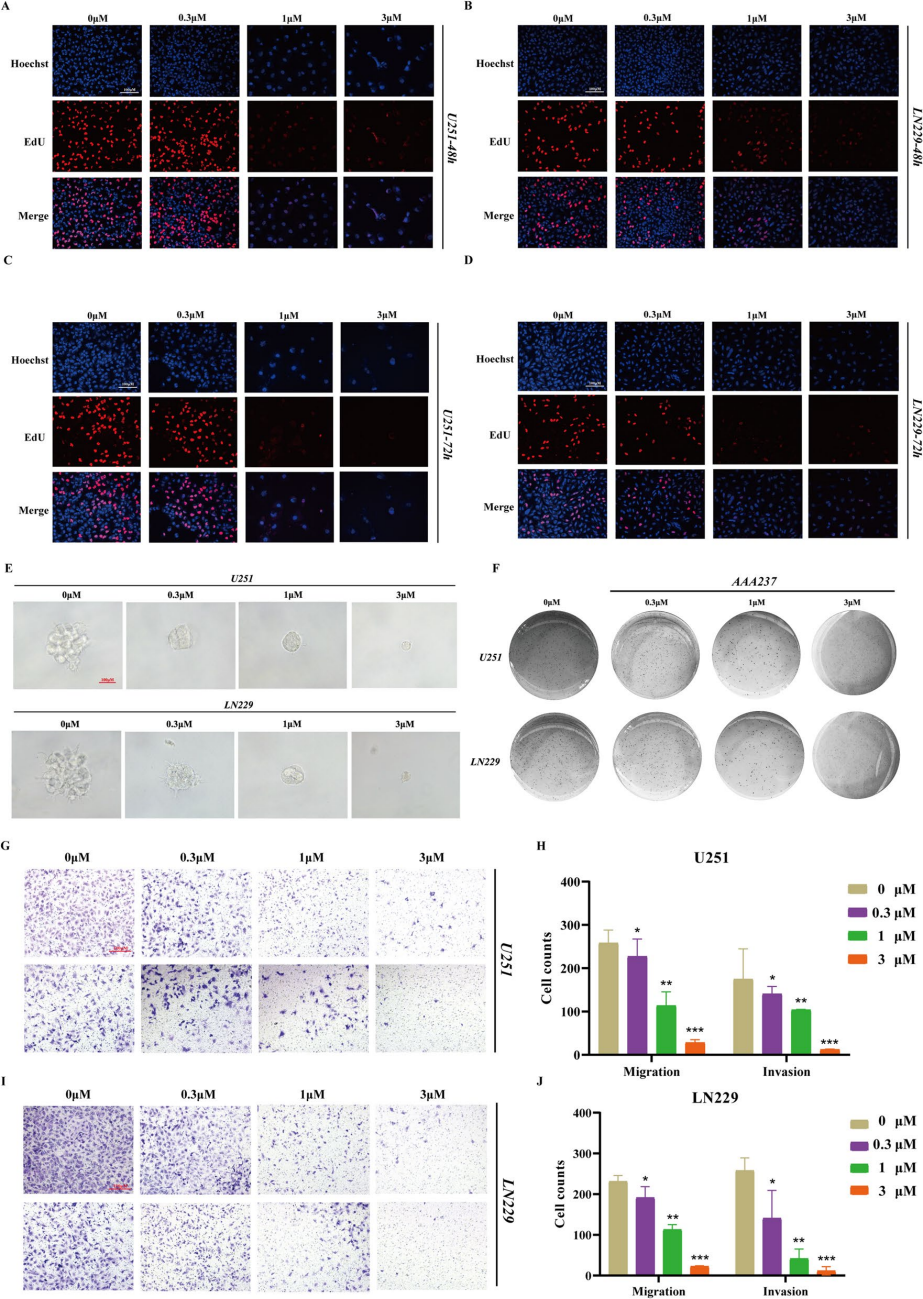
AAA237 dose-dependently inhibited colony formation, migration, and invasion of GBM cells. EdU-DNA synthesis assay shows that AAA237 inhibits DNA synthesis at 48 h in U251 (**A**) and LN229 (**B**) cells. Meanwhile, AAA237 inhibits DNA synthesis at 72 h in U251 (**C**) and LN229 (**D**) cells. Scale bar = 100 μm. **E** Matrigel assay showed that AAA237 reduced the colony formation of U251 and LN229 cells. **F** Soft agar assay showed that AAA237 reduced the colony formation of U251 and LN229 cells. The experiments were performed in triplicate, and scale bar = 100 μm. Transwell assay showed that AAA237 inhibited the migration and invasion of U251 (**G**–**H)** and LN229 (**I**–**J**) cells

The EdU-DNA synthesis test was performed to check the suppressive effects of AAA237 on cell growth. The results of this investigation indicate that the application of AAA237 at doses of 0.3, 1, and 3 μM for periods of 48 and 72 h resulted in a decrease in the proliferation rate of U251 and LN229 cells. Moreover, the inhibitory effect was seen to be dependent on both the dosage and duration of treatment (Fig. 3A–D). In line with these findings, the number of colonies that formed in cells treated with 0.3, 1, or 3 μM AAA237 was less than in the control cells in both Matrigel (Fig. 3E) and soft agar (Fig. 3F). Moreover, the compound AAA237 demonstrated in U251 and LN229 cells, there is a dose-dependent reduction of migration and invasion (Fig. 3G–J). Overall, in a dose-dependent manner, AAA237 demonstrated the ability to inhibit colony formation, migration, and invasion of GBM cells.

### Differential gene expression and enrichment analysis in U251 and LN229 cells treated with AAA237

**Fig. 4:**
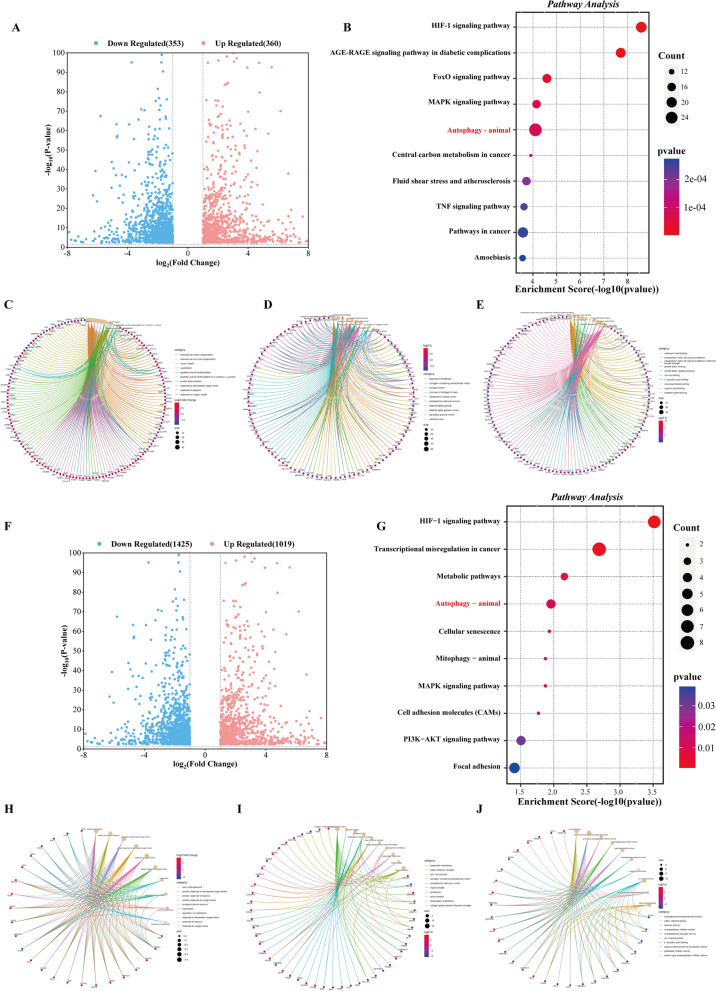
Enrichment analysis and differential gene expression in U251 and LN229 cells treated with AAA237. **A** Volcano plot of differential expression genes in U251 (up-regulated genes are in red; down-regulated genes are in blue (|log2FC|≥ 1 and P value ≤ 0.05). **B** KEGG pathway analysis of differentially expressed genes in U251. **C** The GO enrichment of BP category in U251. **D** The GO enrichment of CC category in U251. **E** The GO enrichment of MF category in U251. **F** Volcano plot of differential expression genes in LN229 (up-regulated genes are in red; down-regulated genes are in blue (|log2FC|≥ 1 and P value ≤ 0.05). **G** KEGG pathway analysis of differentially expressed genes in LN229. **H** The GO enrichment of BP category in LN229. **I** The GO enrichment of CC category in LN229. **J** The GO enrichment of MF category in LN229

To examine the mechanism by which AAA237 affected GBM, an RNA-seq experiment was conducted to identify genes that exhibit differential expression in U251 and LN229 cells following treatment with 0.3 μM AAA237 for a duration of 48 h. In comparison to gene expression in control U251 cells, the cells treated with AAA237 had 360 up-regulated genes and 353 down-regulated genes (Fig. 4A). The findings derived from the KEGG pathway enrichment analysis indicated that the genes exhibited enrichment in pathways that are closely linked to the process of mitophagy, FoxO signaling pathway and MAPK signaling pathway, etc. (Fig. 4B). The findings obtained from the Gene Ontology (GO) enrichment analysis of the DEGs in U251 cells indicated that the genes enriched in the biological processes (BP) category were associated with the control of neuron death and response to hypoxia (Fig. 4C). In the cell components (CC) category, the DEGs were involved in the collagen trimer and vesicle lumen (Fig. 4D). The DEGs identified in the molecular function (MF) category showed involvement in extracellular matrix structural component and growth factor receptor binding (Fig. 4E). Similarly, in comparison to gene expression in control LN229 cells, the cells treated with AAA237 had 1019 up-regulated genes and 1425 down-regulated genes (Fig. 4F). The results derived from the KEGG pathway enrichment analysis indicated that the genes exhibited enrichment in autophagy, MAPK signaling pathway, etc. (Fig. 4G). GO enrichment analysis of the DEGs in U251 cells indicated that the genes enriched in the BP category were associated with cellular response to hypoxia (Fig. 4H). In the CC category, the DEGs were involved in the cell-cell junction (Fig. 4I). The DEGs identified in the MF category showed involvement in channel activity (Fig. 4J).

In comparison to gene expression in control LN229 cells, the cells treated with AAA237 had 1019 up-regulated genes and 1425 down-regulated genes (Fig. 4F). The outcomes of the KEGG pathway enrichment analysis indicated that the genes exhibited enrichment in pathways linked to mitophagy, PI3K-AKT signaling pathway and HIF-1 signaling pathway (Fig. 4G). The findings obtained from the Gene Ontology (GO) enrichment analysis of the differentially expressed genes (DEGs) in LN229 cells demonstrated that the genes enriched in the biological process (BP) category are mostly associated with the cellular response to hypoxia (Fig. 4H). In the CC category, the DEGs were involved in the basement membrane and perikaryon (Fig. 4I). The DEGs under the MF category were shown to be associated with channel activity and L-ascorbic acid binding (Fig. 4J).

### BNIP3 was upregulated in GBM cells treated with AAA237

BNIP3 is a protein related to the BH3-only family, which induces both cell death and autophagy [27]. The evidence suggests that BNIP3 induces cell death through multiple mechanisms [27, 28]. BNIP3 can initiate or enhance autophagy and its variation, mitophagy [29]. We overlapped the DEGs of RNA-seq results in U251 and LN229 after administering AAA237. Surprisingly, BNIP3 was upregulated as a hub gene in both U251 and LN229 cells after the administration of AAA237(the detailed information of overlapped DEGs of RNA-seq in U251 and LN229 cells was shown in Additional file 1: Table S3).

**Fig. 5:**
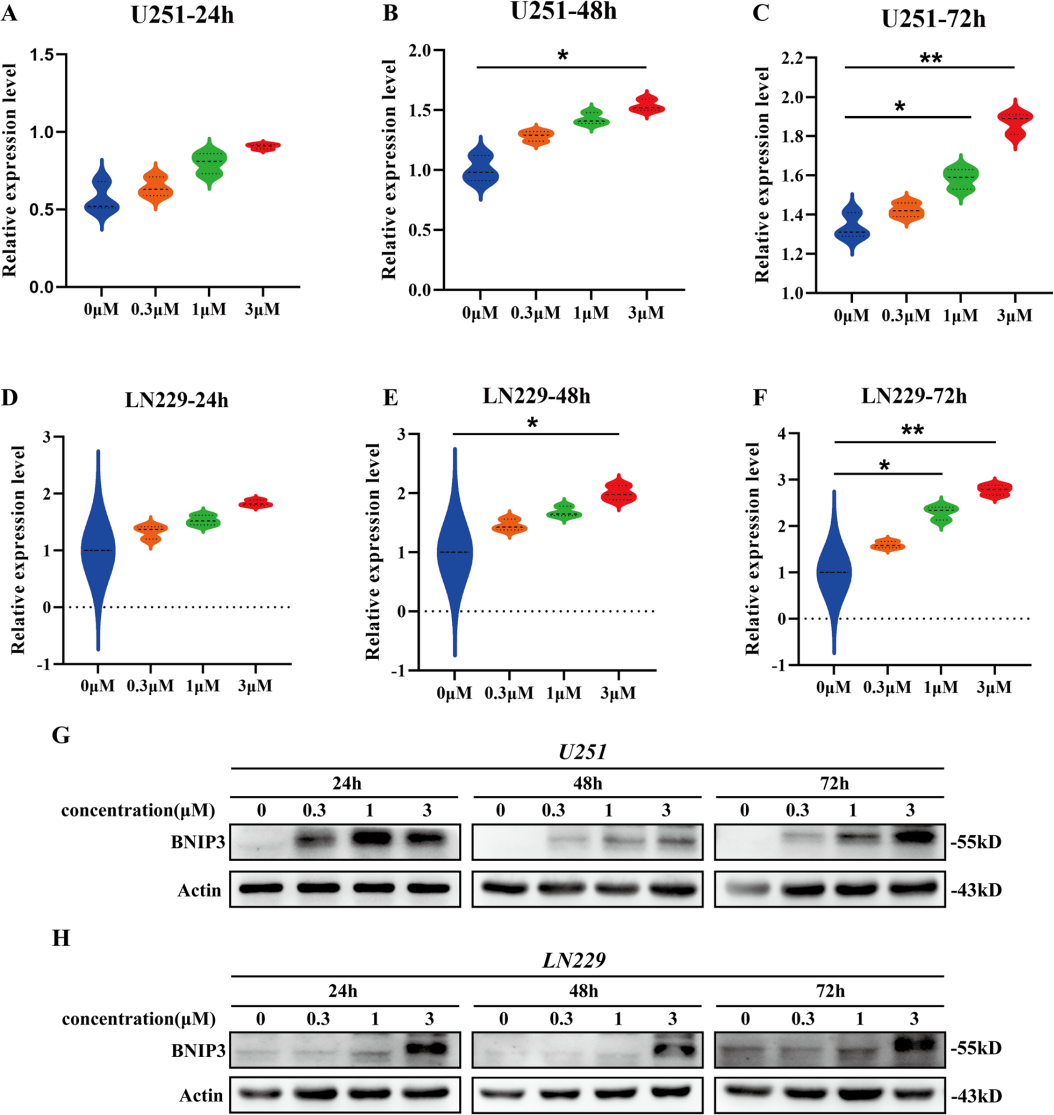
BNIP3 was upregulated as autophagy promotor when AAA237 was administered. **A**–**C** Results from qRT-PCR results showed that AAA237 increased the expression of BNIP3 in a dose-dependent manner in U251. **D**–**F** Results from qRT-PCR results showed that AAA237 increased the expression of BNIP3 in a dose-dependent manner in LN229.The experiments were performed in triplicate, and the data are presented as mean ± SD, *P < 0.05, **P < 0.01 vs. control group. **G** Results of Western blot showed that the protein levels of BNIP3 were increased after treated with AAA237 in U251. **H** Results of Western blot showed that the protein levels of BNIP3 were increased after treated with AAA237 in U251

To confirm the mRNA and protein level of BNIP3 after the administration of AAA237, qRT-PCR and Western blot were performed. Consistent with the RNA-seq results, the mRNA level of BNIP3 was up-regulated as compared to the control group in a dose-dependent manner in both U251 (Fig. 5A–C) and LN229 (Fig. 5D–F). Meanwhile, the protein level of BNIP3 was upregulated in comparison with the control group in both U251 (Fig. 5G) and LN229 (Fig. 5H). Considering that previous studies have shown that BNIP3 has an inhibitory effect on mTOR, Western blot was conducted to verify the effect of BNIP3 on mTOR. The results showed that BNIP3 negatively regulated mTOR (Figure S1). Taken together, the mechanism of AAA237 on GBM was related to the change of BNIP3.

### AAA237 induced autophagy through regulation of the mTOR-mediated pathway

**Fig. 6:**
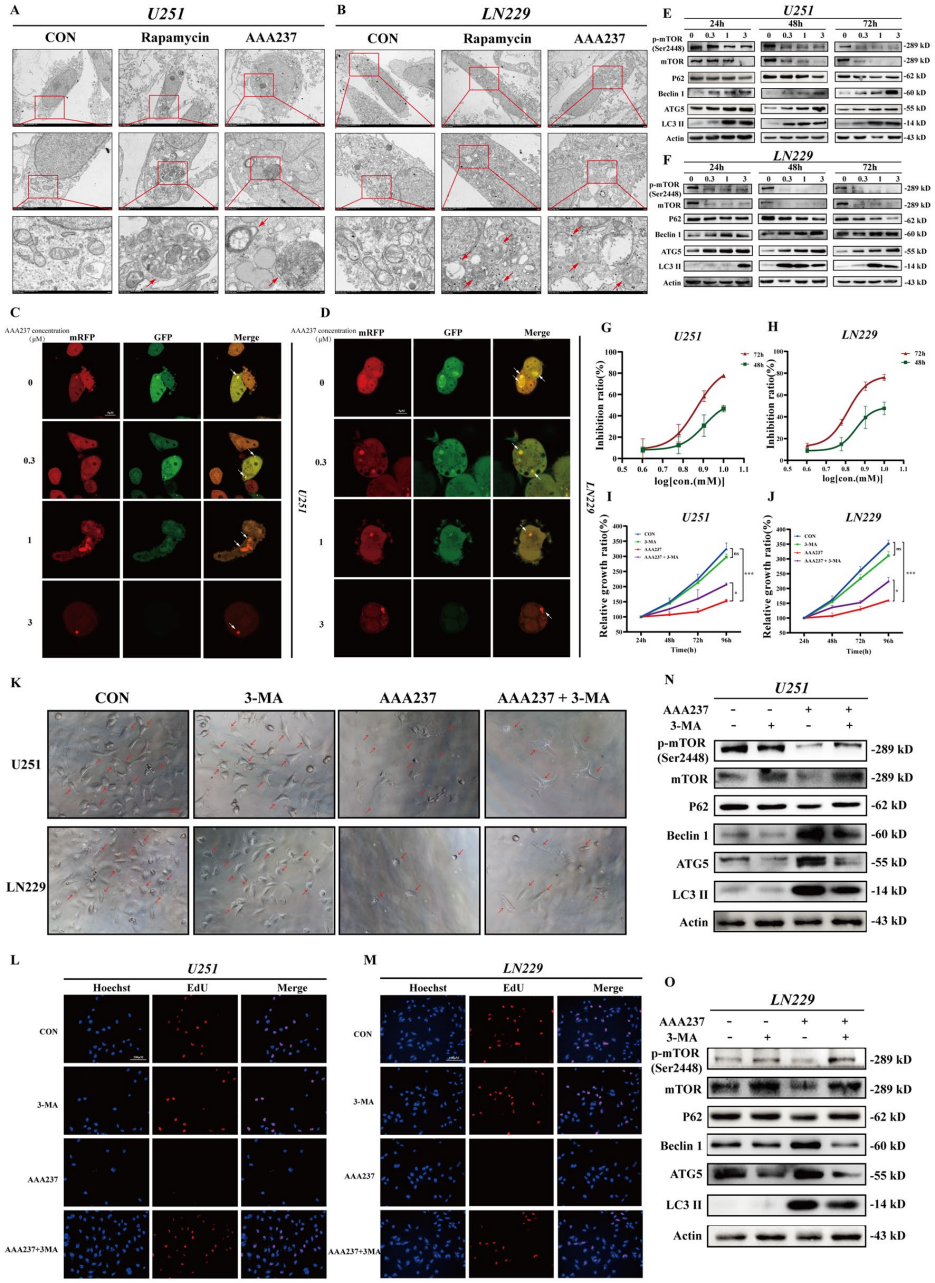
AAA237 induced autophagy through mTOR-mediated pathway regulation. **A** The representative images of transmission electron microscopy (TEM) of U251 cells after treatment of 3 μM AAA237 for 48 h. Scale bar = 500 nm. **B** The representative images of transmission electron microscopy (TEM) of LN229 cells after treatment of 3 μM AAA237 for 48 h. Scale bar = 500 nm. **C** U251 cells with stably expressing mRFP-GFP-LC3 were treated with AAA237 (3 μM) for 48 h and autophagosomes were observed under the fluorescence microscope. Scale bar = 5 μm. **D** LN229 cells with stably expressing mRFP-GFP-LC3 were treated with AAA237 (3 μM) for 48 h and autophagosomes were observed under the fluorescence microscope. Scale bar = 5 μm. **E** Expression of p-mTOR, mTOR, P62, Beclin 1, ATG5 and LC3BII in U251 cells was checked by Western blot under treatment with different concentrations of AAA237(0, 1, 3 and 10 μM) after 24 h, 48 h, 72 h. **F** Expression of p-mTOR, mTOR, P62, Beclin 1, ATG5 and LC3BII in LN229 cells was checked by Western blot under treatment with different concentrations of AAA237(0, 1, 3 and 10 μM) after 24 h, 48 h, 72 h. **G** IC50 of 3-MA on U251. **H** IC50 of 3-MA on LN229. **I** The CCK8 assay was used to show 3-MA could reverse the inhibition of cell proliferation caused by AAA237 in U251. **J** The CCK8 assay was used to show 3-MA could reverse the inhibition of cell proliferation caused by AAA237 in LN229. **K** After incubation with AAA237 and 3-MA for 48 h, the inhibition of cell proliferation caused by AAA237 was reversed. Scale bar = 100 μm. **L**, **M** The EdU-DNA synthesis assay was used to show 3-MA could reverse the inhibition of cell proliferation caused by AAA237 in U251 andLN229. Scale bar = 100 μm. **N**, **O** Expression of p-mTOR, mTOR, P62, Beclin 1, ATG5 and LC3BII in U251 cells was checked by Western blot under treatment with AAA237 and 3-MA

Transmission electron microscopy was used to analyze the morphology of U251 and LN229 cells after they were treated with AAA237 for 48 h in order to ascertain whether the compound might cause autophagy in GBM. As a positive control, rapamycin was also used to incubate the cells. In contrast to the untreated control group, the administration of either rapamycin or AAA237 resulted in the observed development of autolysosomes in U251 (Fig. 6A) and LN229 (Fig. 6B) cells. In addition, we utilized the mRFP-GFP-LC3 indicator system to assess alterations in autophagy flux, to investigate the impact of AAA237 on the dynamic fusion process between autophagosomes and lysosomes. The result showed that AAA237 could induce a significant increase of GFP-LC3 puncta in U251 (Fig. 6C) and LN229 (Fig. 6D) cells, indicating that AAA237 promoted the fusion of lysosomes and autophagosomes, resulting in the formation of autolysosomes. According to these findings, AAA237 increased the union of phagosomes and lysosomes, which resulted in cell autophagy.

mTOR, a serine/threonine kinase, serves as a pivotal regulator of cellular metabolism, exerting control over cell growth and proliferation in response to various stimuli. Notably, the signaling pathway associated with mTOR is disrupted in several clinical disorders [30–32]. mTOR plays a crucial role in regulating autophagy [33, 34]. Considering that the previous transcriptome data suggested that the downstream mechanism may be associated with the PI3K pathway, the mTOR level has been checked in U251 and LN229 cells after the administration of AAA237. Meanwhile, Western blot was carried out to check critical proteins associated with autophagy induced by AAA237. These proteins included p-mTOR, mTOR, Beclin1, P62, LC3B and ATG5. According to the results (Fig. 6E, F), p-mTOR and mTOR were reduced. In contrast, the protein level of LC3-II, a recognized indicator of autophagy, exhibited a dose-dependent elevation subsequent to treatment with escalating doses of AAA237, suggesting that the medication elicited an autophagic reaction. The upregulation of Beclin1 expression resulted in an enhancement in phagophore and autophagosome production. The expression of ATG5 was found to be elevated, leading to an enhanced conversion of LC3-I to LC3-II. Additionally, a drop in p62 levels indicated unimpeded autophagy flow downstream, since p62 serves as a substrate for autophagolysosome destruction. These results above suggested that the induction of autophagy by AAA237 is achieved through the regulation of the mTOR-mediated pathway.

To elucidate how AAA237 activated autophagy, whether 3-Methyladenine (3-MA), an inhibitor of PI3K [35], could reverse AAA237-induced autophagy was examined. 3-MA is widely used as an autophagy inhibitor by inhibiting classIII PI3K [36, 37]. Firstly, the IC_50_ of 3-MA in U251 and LN229 was calculated to choose a concentration with no obvious toxicity and no discernible impact on the cells. The IC_50_ of 3-MA at 48 h in U251 and LN229 was 8.02 mM, 7.136 mM respectively (Fig. 6G, H). Consequently, we employed the 5 mM concentration of 3-MA in subsequent experiments. The CCK-8 assay was used to examine whether 3-MA could reverse the inhibition of cell proliferation caused by AAA237. Interestingly, AAA237-induced cell growth suppression was reversed by 3-MA (Fig. 6I, J). Simultaneously, morphology of GBM cells revealed that 3-MA reversed AAA237’s lethal impact on U251 and LN229 cells (Fig. 6K). Moreover, an EdU-DNA synthesis test was performed to verify the changeover of 3-MA in autophagy. Results showed that 3-MA neutralized the suppression of proliferation in U251 and LN229 cells (Fig. 6L, M). To confirm how 3-MA affected the autophagy flux resulting from AAA237 in GBM, the expression of LC3 and p62 was checked by Western blot. Results showed that the reduction of p-mTOR, mTOR, and p62 was reversed, while 3-MA reversed the increase of Beclin1, ATG5 and LC3II in U251 and LN229 (Fig. 6N, O). To sum up, 3-MA reversed the autophagy caused by AAA237. To summarise the two parts above, AAA237 can cause the onset of autophagy in GBM cells, whereas 3-MA can somewhat reverse the process.

### AAA237 suppressed the growth of LN229 orthotopic tumors

**Fig. 7:**
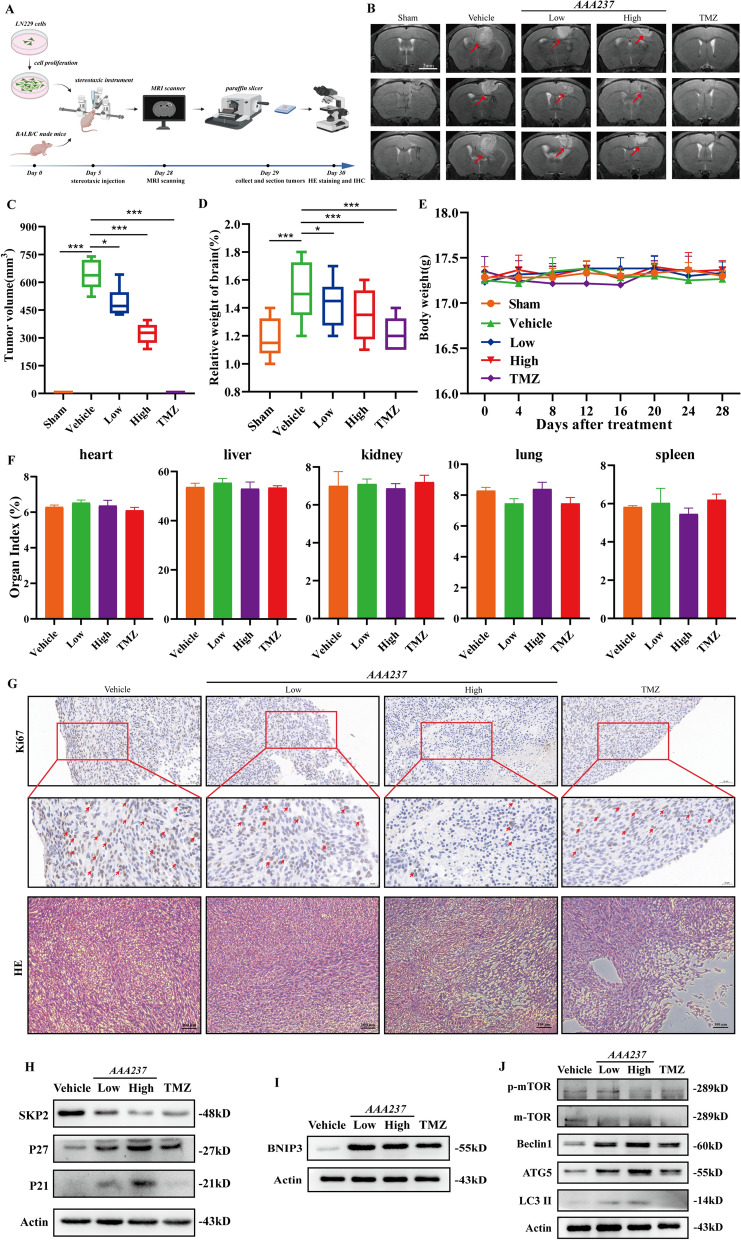
AAA237 inhibited the proliferation of GBM in LN229 orthotopic model. **A** The flow chart of LN229 orthotopic model. **B** Representative MRI images of intracranial tumors from various groups of the LN229 orthotopic model (Scale bar = 2 mm). **C** Tumor volumes in the LN229 orthotopic model. **D** Relative brain weight in the LN229 orthotopic model. **E** Changes in body weight during the AAA237 administration period. **F** AAA237 had no side effects on the major organs of the test mice. **G** H&E staining and immunohistochemical results in brain tissues of GBM orthotopic nude mice model. Scale bar = 50 μm. Data are presented as mean ± SD (n = 6). Statistical significance was determined by one-way ANOVA, ***P < 0.001 vs. Vehicle. **H** Expression of SKP2, P27 and P21 in mouse tumor tissue was checked by Western blot under treatment with low and high concentrations of AAA237 (15 mg/kg and 45 mg/kg). **I** Expression of BNIP3 in mouse tumor tissue was checked by Western blot under treatment with low and high concentrations of AAA237 (15 mg/kg and 45 mg/kg). **J** Expression of p-mTOR, mTOR, Beclin 1, ATG5 and LC3II in mouse tumor tissue was checked by Western blot under treatment with low and high concentrations of AAA237(15 mg/kg and 45 mg/kg)

To investigate the effect of compound AAA237 on tumor growth in vivo, LN229 cells were injected into the brains of mice in an orthotopic model and tumor growth was monitored by magnetic resonance imaging (MRI) (Fig. 7A). The AAA237 (15 and 45 mg/kg) treatment groups effectively retards the progression of tumors in situ (Fig. 7B) which confirmed the in vitro results. The mice in the AAA237 treatment groups had substantially decreased ultimate tumor volume and relative brain weight compared to the animals in the Vehicle group (Fig. 7C, D). Throughout the 21-day period of AAA237 treatment, no discernible instances of weight loss or aberrant behavior were observed (Fig. 7E). Meanwhile, AAA237 had no side effects on the major organs of the test mice (Fig. 7F). The results of H&E staining revealed that the tumor tissue in the Vehicle group exhibited a higher density compared to that in the AAA237 treatment group. Additionally, the protein expression of Ki67 was seen to decrease following the administration of AAA237 (Fig. 7G). Western Blot results of tumor tissues in mouse brain showed that when AAA237 was administered, the level of SKP2 was down-regulated, whereas the levels of SKP2 substrates P27 and P21 were up-regulated (Fig. 7H). Meanwhile, BNIP3 was upregulated in mouse intracerebral tumor tissues upon administration of AAA237 (Fig. 7I). Moreover, autophagy-related markers in mouse intracerebral tumor tissue showed that autophagy occurred when AAA237 was administered (Fig. 7J). Collectively, all the data indicated that the administration of AAA237 effectively suppressed the development of glioma tumors in vivo.

## Discussion

GBM is well recognized as the most widespread and aggressive primary brain tumor. It is characterized by a discouraging prognosis, significant tumor heterogeneity at both intertumoral and intratumoral levels, and a notable absence of efficacious therapeutic interventions [38]. In this study, we confirmed that AAA237 binds to Skp2 biophysically and further investigated the anti-cancer effect of AAA237 in vitro, showing that it inhibited the proliferation, migration and invasion of human GBM cells. Results from the RNA-Seq analysis revealed a differential expression of genes in GBM cells that were subjected to treatment with AAA237, in comparison to the control cells. Notably, the hub gene discovered in this study was BNIP3. In addition, AAA237 could upregulate the level of BNIP3, which was confirmed to activate autophagy via downregulating the mTOR pathway. Further analysis of differential gene enrichment, pathway validation and protein level verification also revealed that AAA237 exerted anti-GBM effects by regulating the mTOR pathway. mTOR plays a crucial role in regulating autophagy. AAA237 had the potential to enhance the dynamic process of autophagosome and lysosome fusion by activating the mTOR pathway. Based on the findings presented, it is plausible to consider AAA237 as a prospective pharmaceutical agent for the treatment of GBM.

Skp2 is classified as an F-box protein, which serves as a constituent of the SKP2-SKP1-Cullin-1-F-box (SCF) ubiquitin ligase tetrameric complex [39]. SKP2 is an oncogene and a regulator of the cell cycle that is responsible for the identification and ubiquitination of phosphorylated proteins involved in cell cycle regulation [39–41]. Seven compounds that inhibit the formation of the Skp2-Skp1 complex have been identified through virtual high-throughput screening and in vitro screening, with SZL-P1-41, in particular, exhibiting potent inhibition of the construction of the Skp2-Skp1 complex in relevant assays and inhibiting tumor growth effectively in vivo [42, 43]. Unfortunately, Chia-Hsin Chan et al. were unable to acquire a single-crystal structure of the small molecule in complex with the Skp2 protein to confirm the actual mode of action, and there is potential for enhancing the anticancer efficacy and pharmacokinetic features of the small molecule [43]. Inspired by this study, our group designed the novel small molecule inhibitor, AAA237, targeting the SKP2-SKP1 protein interaction hotspot. Results from intact cellular thermal shift assay showed that AAA237 binds to Skp2.

BNIP3, a number of BCL2 family, can induce autophagy [27, 44, 45]. BNIP3 is a multifunctional protein localised in the outer mitochondrial membrane that can function as a pro-apoptotic protein or a pro-mitochondrial autophagy receptor. BNIP3 is involved in the nucleation and elongation steps of mitochondrial autophagy and ensures that phagocytic vesicles are recruited to mitochondria by binding to autophagosomal proteins, microtubule-associated proteins 1A/1B light chain 3B (Atg8A or LC3). In addition, BNIP3 binds to the central mitochondrial autophagy protein PTEN-inducible kinase 1 (Pink1), blocking its clearance and inducing mitochondrial autophagy. BNIP3 can induce autophgay via restraing mTOR pathway [46]. Here, BNIP3 was found as the hub gene through overlapping the DEGs of RNA-Seq analysis. Our results revealed that AAA237 up-regulated BNIP3 and suppressed mTOR, which is the novel anti-GBM mechanism and distincted from AAA237-induced senescence in NSCLC [47].

Autophagy, a multistep lysosomal degradation system that promotes nutrient recycling and metabolic adaptability, has been linked to cancer regulation [48, 49]. Autophagy is a multistep process in which damaged or dysfunctional mitochondria are engulfed by phagocytic vesicles to form autophagosomes, which fuse with acidic lysosomes to degrade and circulate the relevant substances within the cell. In cancer, excellent works of autophagy inducers have been demonstrated: BH-3 mimetics, Rapamycin, Curcumin, Quercetin,etc. [50]. Surpringly, AAA237 could induce autophagy in GBM cells via an mTOR-dependent pathway. Furthermore, AAA237 can promote the dynamic process of autophagosome and lysosome fusion. Whereas combination treatment with 3-MA and AAA237 markedly inhibited AAA237-induced autophagy.

In our previous study, the compound AAA237 showed a notable capacity to impede the proliferation of non-small cell lung cancer (NSCLC) cells both in vitro and in vivo. This action was seen to be linked to the interruption of the cell cycle in the G0/G1 phase through the regulation of the Skp2-Cip/Kip and PI3K/Akt-FOXO1 signaling pathways [47]. In that study, it was discovered that AAA237 had anti-tumor properties on NSCLC through the initiation of apoptosis and the induction of senescence resulting from DNA damage. In contrast to our earlier research, we discovered in the current work a brand-new mechanism that AAA237 would activate BNIP3 as the hub gene, through which suppressed mTOR pathway and then induced autophagy in GBM cells. Furthermore, AAA237 has the potential to enhance the dynamic mechanism of autophagosome and lysosome fusion. This mechanism is quite distinguished from previous studies, which is due to the difference in tumor type. To sum up, it might be a potentially effective therapy for GBM.

## Conclusion

Compound AAA237, a novel Skp2 inhibitor, showed potent anti-GBM activity in vitro*.* AAA237 reduced viability and inhibited the proliferation of GBM cells in a dose- and time-dependent manner. AAA237 inhibited colony formation, migration and invasion of GBM cells in a dose-dependent manner. Both mRNA and protein level of BNIP3 was upregulated when treated with AAA237, and the downstream autophagy was activated through mTOR pathway. Autophagy induced by AAA237 was partially reversed when U251 and LN229 cells were treated with AAA237 and 3-MA (an autophagy inhibitor) simultaneously. In vivo, AAA237 suppressed the growth of LN229 orthotopic tumors. Therefore, AAA237, a new Skp2 inhibitor, might be a viable therapeutic drug for the management of GBM (Additional file 2).

### Supplementary Information

**Additional file 1: Figure S1.** BNIP3 siRNA upregulates mTOR levels in GBM cells. (A) Western Blot results showed that the protein level of mTOR was upregulated after BNIP3 was RNA-interfered in U251. (B) Western Blot results showed that the protein level of mTOR was upregulated after BNIP3 was RNA-interfered in LN229.

**Additional file 2: Table S1.** The detailed information of antibodies in this paper. **Table S2.** The sequence of primers involved in this paper. **Table S3.** Overlapped DEGs of RNA-seq in U251 and LN229 cells. (|log2FC|> 2).

## Data Availability

Data will be available on request from the authors.
