# Correction: AAA237, an SKP2 inhibitor, suppresses glioblastoma by inducing BNIP3-dependent autophagy through the mTOR pathway

**DOI:** 10.1186/s12935-024-03473-4

**Published:** 2024-08-17

**Authors:** Yizhi Zhang, Wan Li, Yihui Yang, Sen Zhang, Hong Yang, Yue Hao, Xu Fang, Guanhua Du, Jianyou Shi, Lianqiu Wu, Jinhua Wang

**Affiliations:** 1 The State Key Laboratory of Bioactive Substance and Function of Natural Medicines, Beijing, 100050 China; 2 Key Laboratory of Drug Target Research and Drug Screen, Institute of Materia Medica, Chinese Academy of Medical Science and Peking Union Medical College, Beijing, 100050 China; 3 Department of Pharmacy, Personalized Drug Therapy Key Laboratory of Sichuan Province, Sichuan Academy of Medical Sciences & Sichuan Provincial People’s Hospital, School of Medicine, University of Electronic Science and Technology of China, Chengdu, 610072 Sichuan China; 4 Department of Pharmacology, Institute of Materia Medica, Chinese Academy of Medical Science and Peking Union Medical College, Beijing, 100050 China

**Correction: Cancer Cell International (2024) 24:69** 10.1186/s12935-023-03191-3

In this article [1], the wrong figure appeared as Fig. 1A, Fig. 4F and Fig. 6K; the corrected figures (Figs. 1, 4, 6) are given in this correction.

**Fig. 1:**
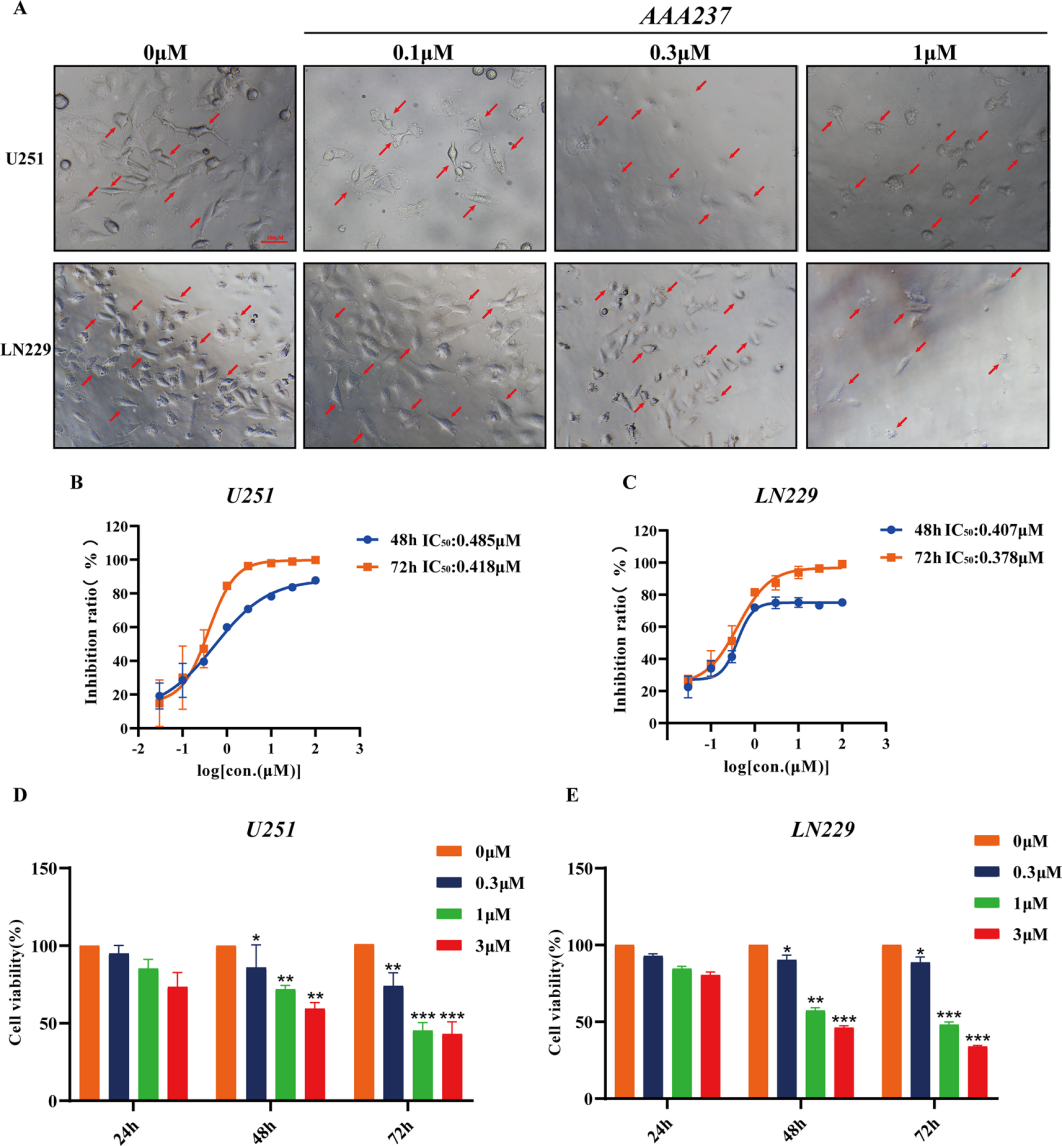
AAA237 suppressed viability and inhibited the proliferation of GBM cells in a dose- and time-dependent manner. **A** After incubation with different concentrations (0, 0.1, 1 and 3 μM) of AAA237 for 48 h, the changes in cell morphology were imaged. Scale bar = 100 μm. IC50 of AAA237 on U251 (**B**) and LN229 cells (**C**) at 48 and 72 h. CCK8 assay shows that AAA237 inhibits proliferation of U251 (**D**) and LN229 (**E**) cells

**Fig. 4:**
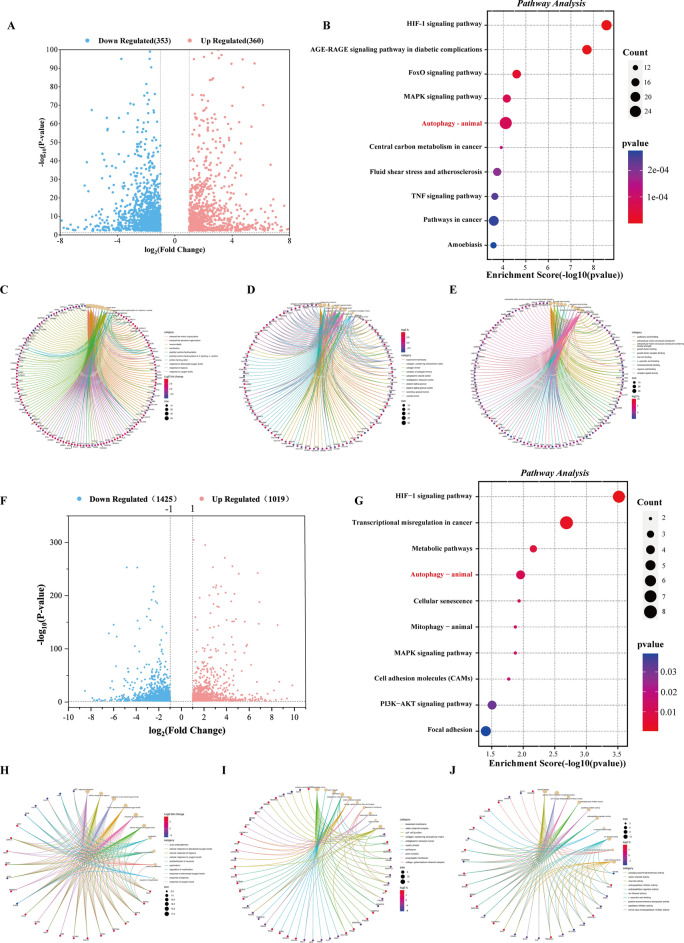
Enrichment analysis and differential gene expression in U251 and LN229 cells treated with AAA237. **A** Volcano plot of differential expression genes in U251 (up-regulated genes are in red; down-regulated genes are in blue (|log2FC| ≥ 1 and P value ≤ 0.05). **B** KEGG pathway analysis of differentially expressed genes in U251. **C** The GO enrichment of BP category in U251. **D** The GO enrichment of CC category in U251. **E** The GO enrichment of MF category in U251. **F** Volcano plot of differential expression genes in LN229 (up-regulated genes are in red; down-regulated genes are in blue (|log2FC| ≥ 1 and P value ≤ 0.05). **G** KEGG pathway analysis of differentially expressed genes in LN229. **H** The GO enrichment of BP category in LN229. **I** The GO enrichment of CC category in LN229. **J** The GO enrichment of MF category in LN229

**Fig. 6:**
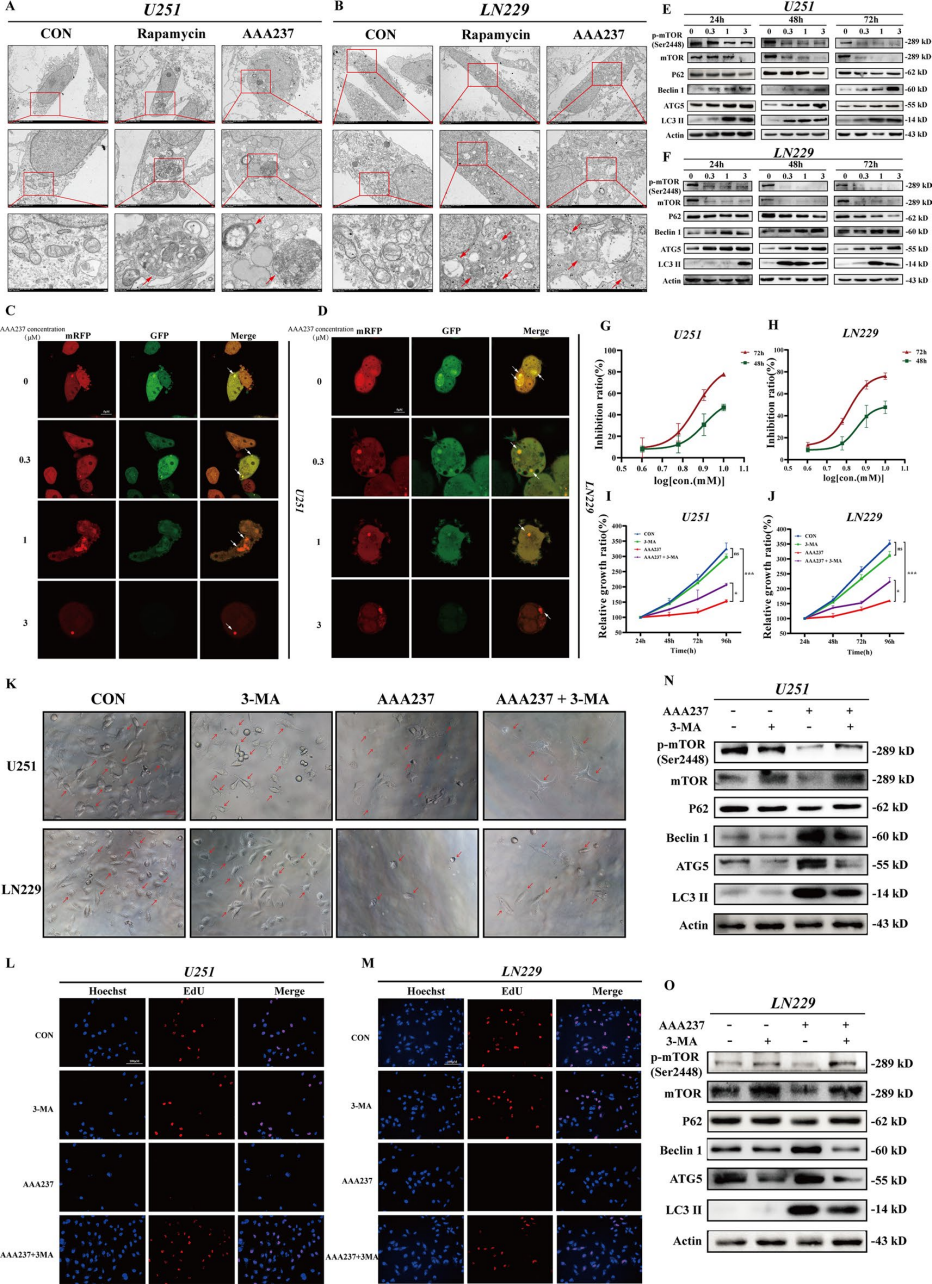
AAA237 induced autophagy through mTOR-mediated pathway regulation. **A** The representative images of transmission electron microscopy (TEM) of U251 cells after treatment of 3 μM AAA237 for 48 h. Scale bar = 500 nm. **B** The representative images of transmission electron microscopy (TEM) of LN229 cells after treatment of 3 μM AAA237 for 48 h. Scale bar = 500 nm. **C** U251 cells with stably expressing mRFP-GFP-LC3 were treated with AAA237 (3 μM) for 48 h and autophagosomes were observed under the fluorescence microscope. Scale bar = 5 μm.** D** LN229 cells with stably expressing mRFP-GFP-LC3 were treated with AAA237 (3 μM) for 48 h and autophagosomes were observed under the fluorescence microscope. Scale bar = 5 μm. **E** Expression of p-mTOR, mTOR, P62, Beclin 1, ATG5 and LC3BII in U251 cells was checked by Western blot under treatment with different concentrations of AAA237 (0, 1, 3 and 10 μM) after 24 h, 48 h, 72 h. **F** Expression of p-mTOR, mTOR, P62, Beclin 1, ATG5 and LC3BII in LN229 cells was checked by Western blot under treatment with different concentrations of AAA237 (0, 1, 3 and 10 μM) after 24 h, 48 h, 72 h. **G** IC50 of 3-MA on U251. **H** IC50 of 3-MA on LN229. **I** The CCK8 assay was used to show 3-MA could reverse the inhibition of cell proliferation caused by AAA237 in U251. **J** The CCK8 assay was used to show 3-MA could reverse the inhibition of cell proliferation caused by AAA237 in LN229. **K** After incubation with AAA237 and 3-MA for 48 h, the inhibition of cell proliferation caused by AAA237 was reversed. Scale bar = 100 μm. **L**, **M** The EdU-DNA synthesis assay was used to show 3-MA could reverse the inhibition of cell proliferation caused by AAA237 in U251 andLN229. Scale bar = 100 μm. **N**, **O** Expression of p-mTOR, mTOR, P62, Beclin 1, ATG5 and LC3BII in U251 cells was checked by Western blot under treatment with AAA237 and 3-MA
