# Supplementary figures and images for: AAA237, an SKP2 inhibitor, suppresses glioblastoma by inducing BNIP3-dependent autophagy through the mTOR pathway
Source: Cancer Cell Int. 2024 Feb 10;24:69. doi: 10.1186/s12935-023-03191-3 (PMC10859026; doi:10.1186/s12935-023-03191-3)

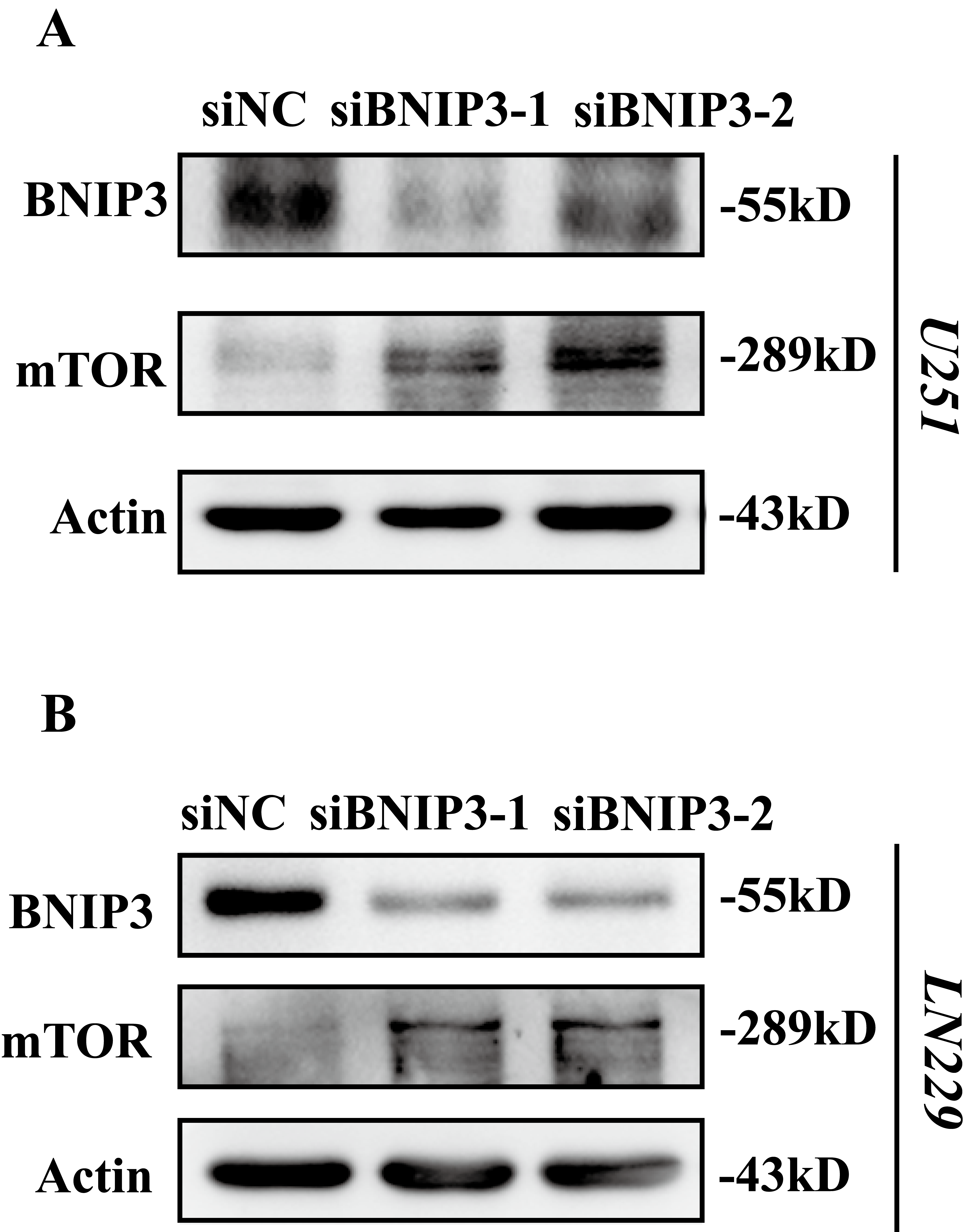

Supplement: Supplementary file 1 — Additional file 1: Figure S1. BNIP3 siRNA upregulates mTOR levels in GBM cells. (A) Western Blot results showed that the protein level of mTOR was upregulated after BNIP3 was RNA-interfered in U251. (B) Western Blot results showed that the protein level of mTOR was upregulated after BNIP3 was RNA-interfered in LN229. [file 12935_2023_3191_MOESM1_ESM.tif]
