# Supplementary material for: AAA237, an SKP2 inhibitor, suppresses glioblastoma by inducing BNIP3-dependent autophagy through the mTOR pathway
Source: Cancer Cell Int. 2024 Feb 10;24:69. doi: 10.1186/s12935-023-03191-3 (PMC10859026; doi:10.1186/s12935-023-03191-3)
Supplement: Supplementary file 2 — Additional file 2: Table S1. The detailed information of antibodies in this paper. Table S2. The sequence of primers involved in this paper. Table S3. Overlapped DEGs of RNA-seq in U251 and LN229 cells. (|log2FC|> 2). [file 12935_2023_3191_MOESM2_ESM.docx]

**AAA237, an SKP2 inhibitor, suppresses glioblastoma by inducing BNIP3-dependent autophagy through the mTOR pathway**

**Table S1.** The detailed information of antibodies in this paper.

| **Antibody** | **Dilution rate** | | **Catalogue** | **Company** |
| --- | --- | --- | --- | --- |
| SKP2 | | 1:1000 | 4313S | Cell Signaling Technology |
| P27  P21  Actin  BNIP3  p-mTOR  mTOR  P62  Beclin1  ATG5  LC3Ⅱ | | 1:1000  1:1000  1:3000  1:1000  1:1000  1:1000  1:1000  1:1000  1:1000  1:1000 | 3686S  2947S  20536-1-AP  44060S  5536S  2983S  5114S  3738S  9980S  13118S | Cell Signaling Technology  Cell Signaling Technology  Proteintech  Cell Signaling Technology  Cell Signaling Technology  Cell Signaling Technology  Cell Signaling Technology  Cell Signaling Technology  Cell Signaling Technology  Cell Signaling Technology |

**Table S2.** The sequence of primers involved in this paper.

| Primer | Sequence |
| --- | --- |
| BNIP3-Forward  BNIP3-Reverse  GAPDH-Forward | CAGGGCTCCTGGGTAGAACT  CTACTCCGTCCAGACTCATGC  GGAGCGAGATCCCTCCAAAAT |
| GAPDH-Reverse | GGCTGTTGTCATACTTCTCATGG |

**Table S3.** Overlapped DEGs of RNA-seq in U251 and LN229 cells. (|log2FC|＞2)

| Gene | U251 log2FC | LN229 log2FC |
| --- | --- | --- |
| SLAMF9 | 8.0615 | 9.8141 |
| SPINK1  LINC00520  SLAMF7  TREM1  AOX1  CPA4  CXCL8  CA9  ALK  SERPINB7  TMEM158  NDRG1  INHBA  ALDOC  HILPDA  **BNIP3**  ADM  MAP3K7CL  TRAF1  ZP1  PTPRR  COL13A1  ADPRHL1  SYTL2  MAFF  P4HA1  TFRC  IL7R  ENO2  ATF3  FOSB  LUAT1  PPP1R3C  TMEM255B  KCNMA1  LOXL2  PDK1  TMEM45A  PTPRZ1  CHL1  SERPINA5  NQO1  PDE7B  ACKR1  UNC5C  PIK3R3  IL1R1  NNMT  SLC7A11  LRP1B | 5.9908  5.8381  8.0615  4.5821  4.4901  4.2646  4.0786  3.9776  3.891  3.8064  3.786  3.6719  3.4272  3.3139  3.2579  2.4538  3.1724  3.0849  3.0086  2.9933  2.9481  2.9301  2.8934  2.7528  2.6596  2.4959  2.4958  2.4934  2.4508  2.3279  2.2752  2.2421  2.1888  2.162  2.1248  2.0909  2.0479  2.0399  -4.4536  -4.2235  -3.7076  -3.4111  -3.3342  -2.9076  -2.870  -2.6641  -2.579  -2.384  -2.3483  -2.2179 | 5.4367  5.3219  9.8141  5.0552  6.6851  8.5132  3.4436  8.6612  6.2841  4.2752  6.9084  4.2578  3.5369  4.280  6.1627  5.0827  4.5605  4.0034  3.0835  7.6292  5.2022  5.2899  8.1812  2.4543  2.0753  4.515  3.1632  3.3522  5.5611  2.7493  2.7371  5.3408  3.1312  4.1023  2.0674  2.0371  4.7667  4.5541  -3.0948  -6.1299  -3.5599  -2.8697  -7.4313  -3.1592  -3.2043  -3.3504  -2.1338  -3.3938  -4.6691  -2.7807 |
